# Chromosomal structural analysis in carcinoma of the gallbladder

**DOI:** 10.1186/1477-7819-10-198

**Published:** 2012-09-25

**Authors:** Ruhi Dixit, Prabhat Kumar, Ratnakar Tripathi, Somprakash Basu, Rajnikanth Mishra, VK Shukla

**Affiliations:** 1 Department of General Surgery Institute of Medical Sciences, Banaras Hindu University, Varanasi, 221 005, India; 2 Department of Zoology, Faculty of Science, Banaras Hindu University, Varanasi, 221 005, India

**Keywords:** Lactate dehydrogenase (LDH), Superoxide dismutase (SOD), Catalase, Carcinoma of the gallbladder

## Abstract

**Background:**

The etiopathogenesis of gallbladder cancer is still unknown. Both environmental and patient factors have been incriminated in its cause. That it is found in pockets of epidemiological distribution raises an issue of genetic changes associated with it. The aim of this study was to find out the chromosomal changes in gallbladder cancer.

**Methods:**

Lymphocyte cell culture was carried out on blood of gallbladder cancer patients to determine chromosomal banding abnormalities. Native PAGE was also evaluated to analyze lactate dehydrogenase (LDH), superoxide dismutase (SOD) and catalase enzyme activity from the same blood of gallbladder cancer patients.

**Results:**

Out of 30 gallbladder cancer patients, 4 male showed breakage on the long arm of chromosome 1 while only one male patient showed the translocation from the long arm of chromosome 4 to the long arm of chromosome 6 in a male patient.

**Conclusion:**

The aberrations found in our study may suggest underlying genetic predisposition for the development of gallbladder cancer. They can act as a marker for gallbladder cancer, which needs further study.

## Background

Carcinoma of the gallbladder (CaGB) is the most common malignancy of the biliary tract and represents 2% of all cancers. It is the third most common gastrointestinal malignancy in northern India [1]. Although a lethal disease and known for decades, its exact etiopathogenesis still eludes physicians. Its cause is thought to be multifactorial with involvement of both environmental and patient factors, such as chronic cholecystitis, gallstones, choledochal cysts, female gender, age and exposure to carcinogens; but a definite cause-effect relationship is yet to be established [2]. That it is found in pockets of epidemiological distribution raises an issue of genetic or chromosomal factors associated with it. The exact sequence of the molecular changes that lead to neoplastic transformation in the gallbladder epithelium remains uncertain. There is limited information available about the molecular abnormalities involved in its pathogenesis. However, few reports described a genetic model for carcinogenesis during the development and progression of gallbladder cancer [3]. Previous studies have identified the presence of regions of frequent allele loss, involving loss of heterozygosity (LOH) in the chromosomes of gallbladder cancer patients [3], especially in the 3p, 8p, 9q and 22q chromosomal regions [4].

Multiple pathways control the accurate duplication and distribution of DNA to progeny cells while other pathways control regulatory modifications of DNA during normal development. These are known as the genetic stability functions [5]. Genetic instability is considered to be the root cause of the phenotypic and genotypic variation between the different cell populations of a tumor [6]. We hypothesized that CaGB in Indian patients may be associated with chromosomal changes, which may lead to a better understanding of a correlation between the two. The aim of the present work is to find out the chromosomal changes in gallbladder cancer and to identify the most consistent changes involved in the pathogenesis of gallbladder cancer.

## Methods

### Subjects

Thirty newly diagnosed cases of gallbladder cancer, which had not yet undergone any treatment in the form of surgery, chemotherapy and radiotherapy, were included consecutively in the study from September 2007 to July 2009 after obtaining informed consent from the patients. This was a single institution study. Histopathological or cytological diagnosis was obtained before inclusion in the study. This study was approved by the institution’s ethics committee.

A venous blood sample (2 mL) was collected from each patient using heparin as an anticoagulant, and chromosomal analysis was carried out in the Biochemistry and Molecular Biology lab, Department of Zoology, Faculty of Sciences of our university.

### Lymphocyte culture

The collected blood samples were centrifuged at 3,000 rpm for five minutes. Pellets from the centrifuged blood were discarded and the “buffy coat” was taken for lymphocyte culture. Lymphocyte culture was set in 7 ml of RPMI (Roswell Park Memorial Institute) 1640 media with 10% FBS (fetal bovine serum) as the nutrient in a sterile culture tube. A total of 100 μl of PHA-M (Phytohemagglutinin-M-5 mg/ml-Himedia) was added in the tube as a mitogen with 0.5 ml of lymphocyte culture. The culture was incubated for 72 hours in a CO_2_ incubator at 37°C with 5% CO_2._

A total of 4 μl of colchicine (1 mg/ml; Himedia) was added before harvesting at the 69^th^ hour to arrest the chromosome in the metaphase stage. The culture was harvested at the 72^nd^ hour and centrifuged at 2,200 rpm for five minutes and the supernatant was discarded. Pre-warmed (37°C) hypotonic solution (0.56% of KCl-Merck) was added to the pellets and kept in an incubator for 20 minutes at 37°C to swell the cells and spread the chromosomes. After hypotonic treatment, it was centrifuged again at 2,200 rpm for five minutes; the supernatant was discarded and 5 ml of Carney’s fixative (3:1, methanol: glacial acetic acid) was added drop by drop. The pellet was agitated vigorously and resuspended in fixative. This was repeated three times. Finally, the pellet was resuspended in 0.5 ml of fixative. Suspension was splashed (forcing the chromosomes into a single plane) on chilled slides and was flame dried.

Slides with good chromosome preparation were selected. One ml of trypsin stock solution (30 mg/ml) was made up to 50 ml with 0.9% sodium chloride in a coupling jar (pH 7.5 to 7.8). The slides were stained in Giemsa for three to five minutes, rinsed in H_2_O and observed under a microscope.

Native polyacrylamide gel (8%) electrophoresis was performed to analyze the lactate dehydrogenase (LDH), superoxide dismutase (SOD) and catalase enzyme activity in the blood of the gallbladder cancer patients. Electrophoresis was carried out at 4°C for three hours, applying a voltage of 10 mV. The gel was stained for a specific enzyme, that is, LDH, catalase and SOD.

a) LDH activity: To analyze the activity of LDH, the gels were stained with LDH specific stain. LDH specific staining was done according to the method of Worthington with modification [7]. The staining solution of LDH consists of 0.1 M Tris-Cl (2-amino-2-hydroxymethyl-propane-1,3-diol- HCl) buffer (pH 8.4), 1 mg/ml nicotinamide adenine dinucleotide (NAD^+^), 0.5 mg/ml nitroblue tetrazolium (NBT), 0.1 mg/ml phenozine methosulphate (PMS) and 0.05 M lithium lactate.

b) Catalase activity: The activity of catalase in gallbladder cancer was performed by staining the gel with catalase specific stain. The stain contains 0.03% hydrogen peroxide, 0.2% potassium ferricynide and 0.2% ferric chloride [8].

c) SOD Activity: Similarly, the SOD activity in the blood was analyzed by staining the gels with SOD specific stain. The stain contained phosphate buffer 0.1 M (pH 7.4), 2.3 mM NBT (nitroblue tetrazolium), 28 μM, riboflavin and 28 mM N N N N’ tetramethyelenediamine (TEMED) [9].

## Results

**Figure 1:**
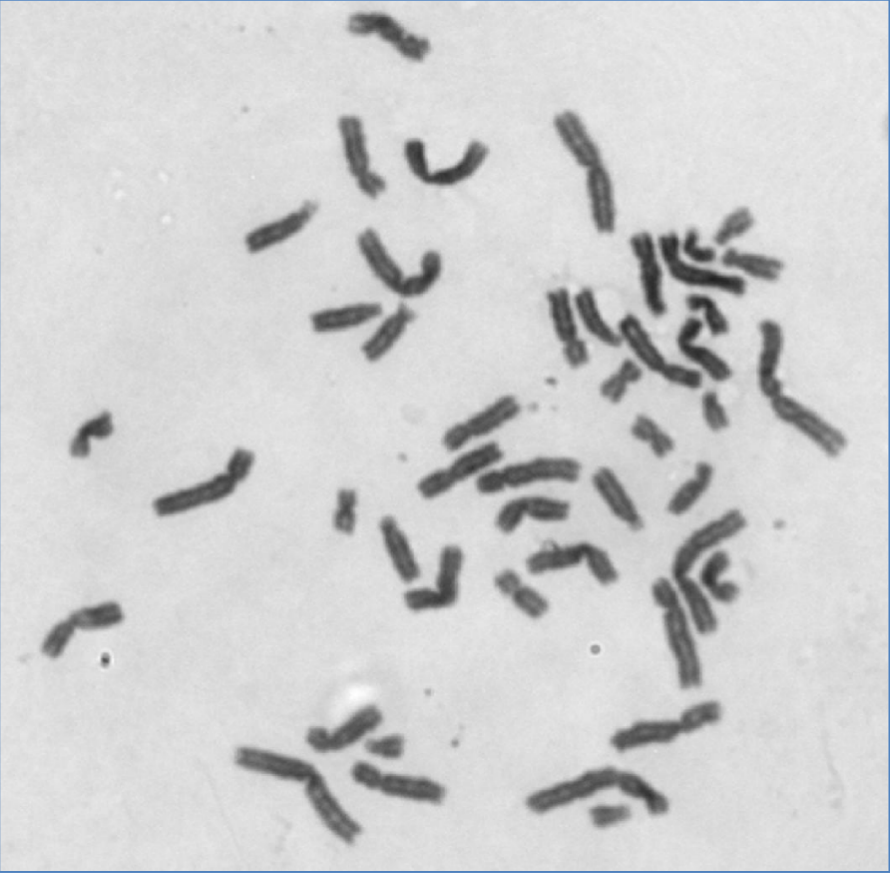
The metaphase plate shows the chromosomes in a normal male. It shows the all 44, XY chromosomes.

**Figure 2:**
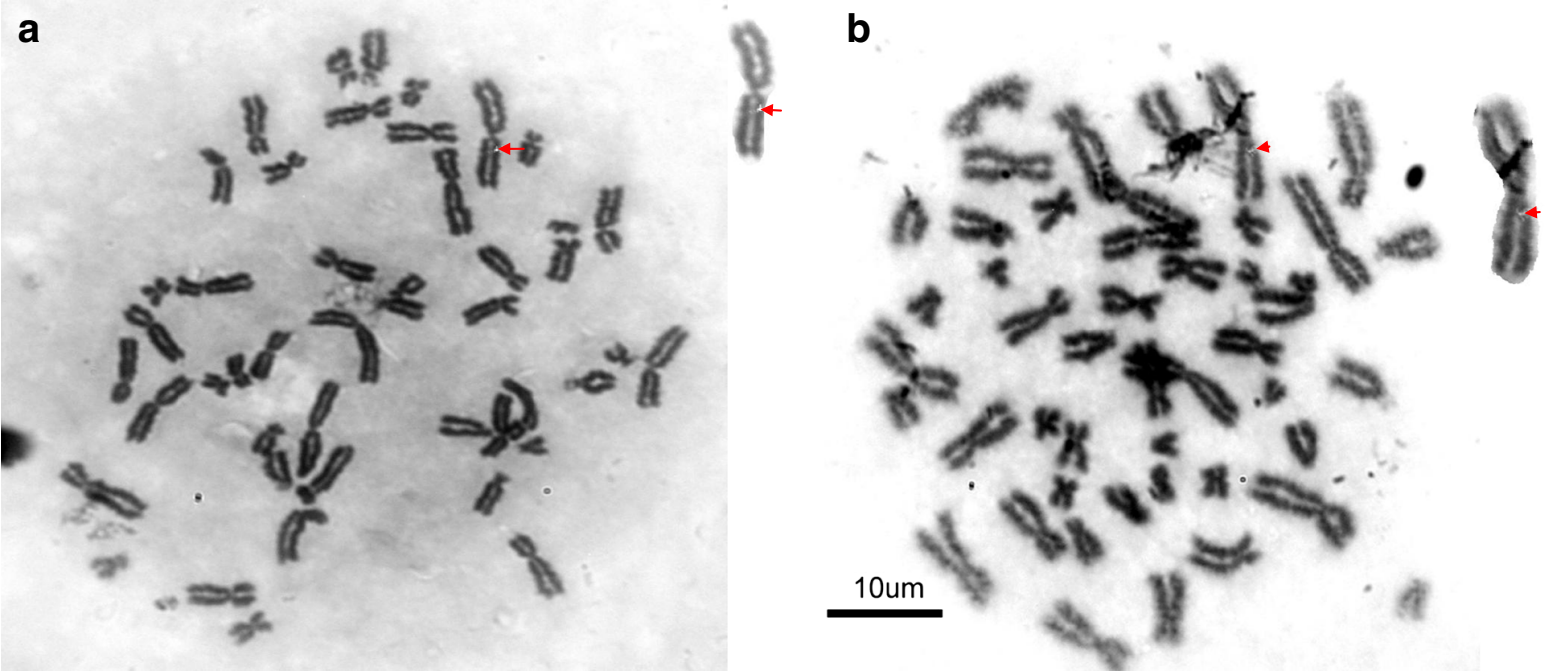
a. Figure shows the breakage of long arm of chromosome 1 in a male patient. b. Image showing the breakage of the long arm of chromosome 1 in a male patient.

**Figure 3:**
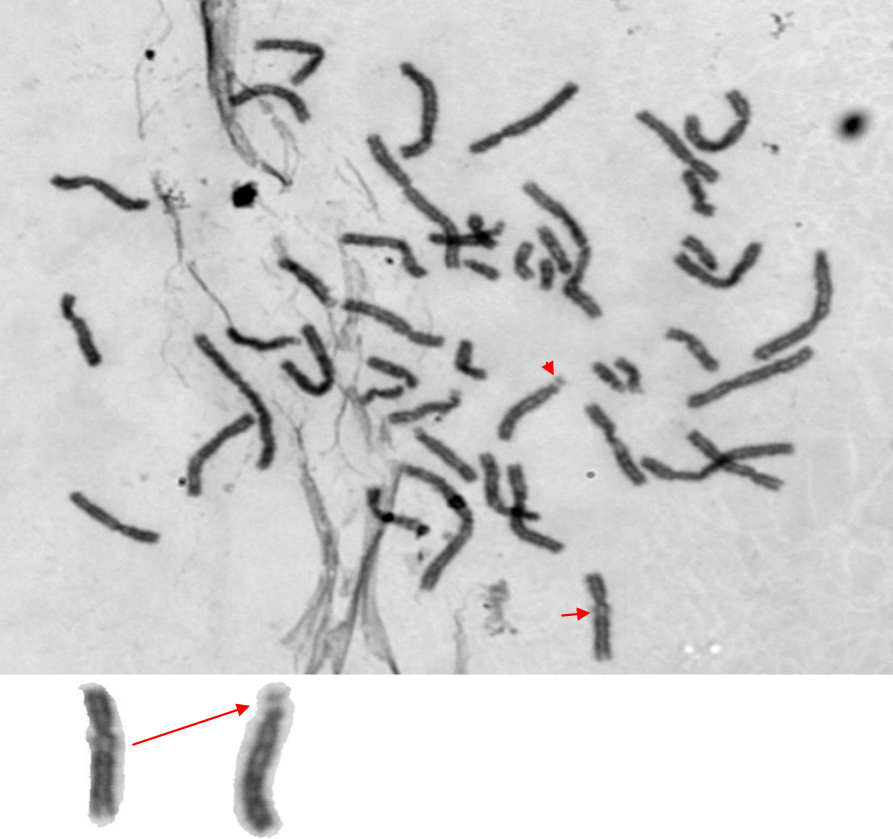
This figure shows the translocation from the long arm of chromosome 4 to the long arm of chromosome 6 in a male patient.

The study was performed with 30 cases of histologically confirmed carcinoma of the gallbladder. The mean age of the patients was 49.9 ± 10.59 years. Out of 30 selected patients 23.3% (7) were males and 76.7% (23) were females. The sex ratio was 3.3:1 (female:male). The peripheral lymphocyte metaphase plate stained with Giemsa stain showed a male metaphase plate carrying 44, XY but no chromosomal aberration was detected (Figure 1). A normal pattern of chromosome distribution was found on the karyotype. Out of 30 patients, 4 males showed breakage at the long arm of chromosome 1 (Figure 2a, b). One male patient showed translocation from the long arm of chromosome 4 to the long arm of chromosome 6 (Figure 3). Patients having such aberrations were histologically proven to have adenocarcinoma. Five patients who showed chromosomal aberrations all had well differentiated tumors.

### Activity of LDH and antioxidant in gallbladder cancer

**Figure 4:**
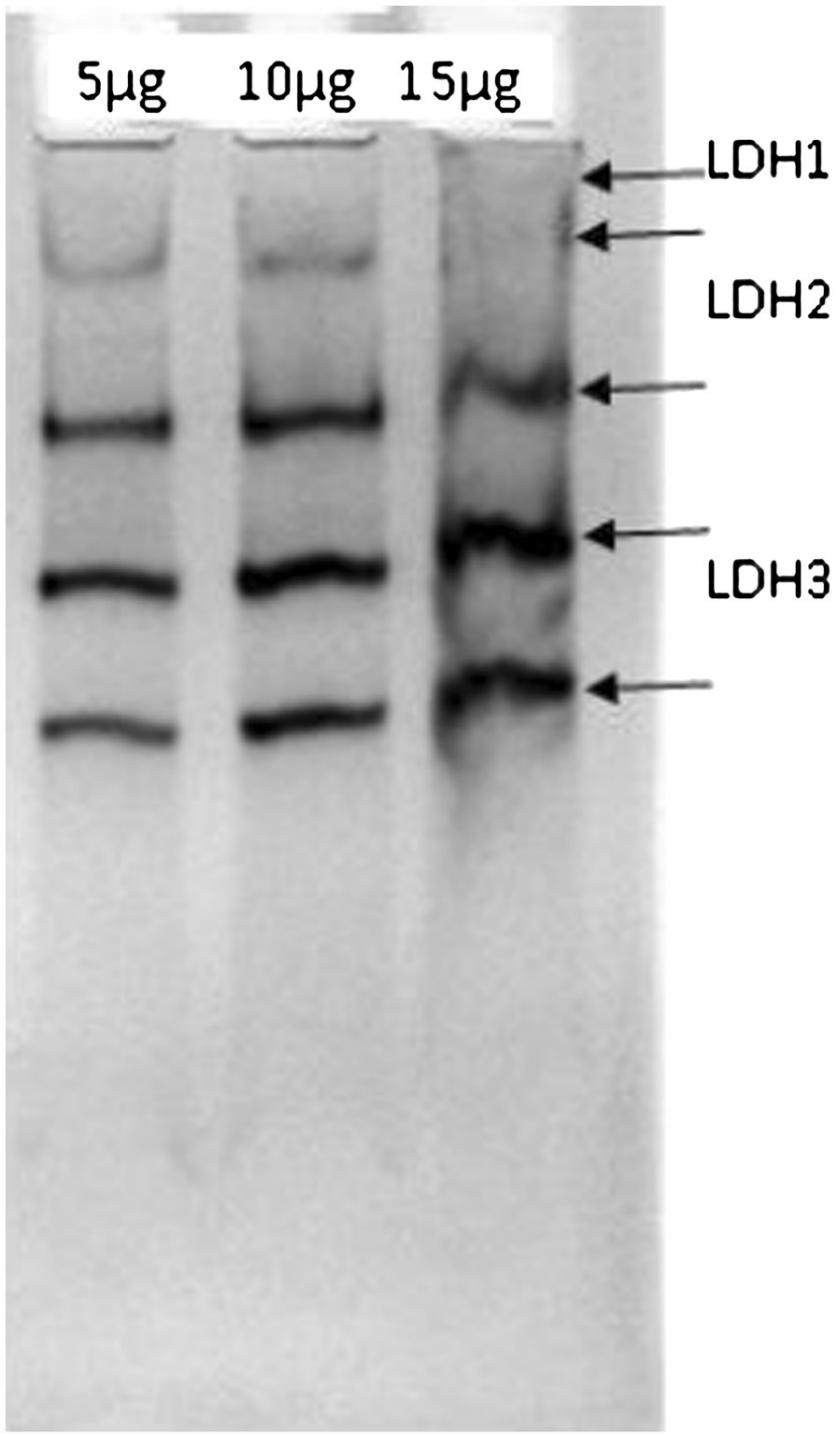
Native PAGE (8%) showing enzyme activity of lactate dehydrogenase (LDH) in the serum of gallbladder cancer patients.

The serum showed high activity of LDH2 in gallbladder cancer, which is the prominent form in serum. Concentrations of LDH3, LDH4 and LDH5 were also elevated in gallbladder cancer (Figure 4). Increased activity of antioxidant enzymes, such as catalase, was observed while the activity of Mn-Zn-SOD was reduced in gallbladder cancer.

## Discussion

Carcinoma of the gallbladder is a lethal disease of the biliary tract [6]. The epidemiological distribution of this disease suggests that the presence of genetic, nutritional and environmental factors may play roles in the etiopathogenesis of the malignancy [10]. The incidence rate of gallbladder cancer varies and is confined to selected pockets in India, Korea, Japan, Czech Republic, Slovakia, Spain, Colombia, Chile, Peru, Bolivia and Ecuador [10]. This wide difference in incidence rate indicates a probable strong association of this malignancy with genetic factors that are common to the affected populations.

Acquired or inherent genetic instability in normal cells causes mutational events that result in neoplastic transformation and provide such cells with a selective growth advantage over normal cells. These genetic instabilities can be traced early by analysis of the chromosomal banding pattern in the cancer patients, which addresses the genetic instabilities associated with various cancers [11]. It was hypothesized that the chromosomal aberrations may be evident in any type of cancer [11] and it was proved in case of endometrial carcinoma where 4% metaphase plates of peripheral blood lymphocytes were positive for the chromosome breakage. Similarly, peripheral blood cultures of patients with breast cancer, malignant melanoma, colon cancer, renal cell carcinoma or lung cancer have shown simple chromosomal breaks that were also marker chromosomes for their respective tumors [12]. Therefore, peripheral blood lymphocyte cultures can be a useful tool for demonstration of simple chromosomal lesions of cancer cells. The specific chromosomal anomalies that can be easily picked up in peripheral blood can act as an important marker for the disease.

In the present study, we have examined the peripheral blood of gallbladder cancer patients for chromosomal banding abnormalities on lymphocytes cell culture. Out of 30 cases, 5 (16.6%) cases showed chromosomal aberrations. The simple aberrations were observed in four patients. These aberrations were confined on chromosome 1’s long arm. Another aberration was a translocation from the long arm of chromosome 4 to the long arm of chromosome 6. An interesting observation is that all the aberrations were present in the male karyotype. The cause of this breakage in chromosomes of gallbladder patients is unknown, but its association with gallbladder carcinoma is suggested. It may be due to environmental effects, infections and inflammation but further work is needed to establish a definite relation between these aberrations and gallbladder cancer. The prevalence of aberrations in our study is quite high, that is, 16.6%. So, we can presume that this will help to find the correlation between such aberrations and gallbladder cancer. Further research will clarify an understanding of the role of chromosomal anomalies in CaGB.

Previous reports had attempted to establish the significance of chromosomal structural changes in various cancer types. But this kind of study has not been reported in gallbladder cancer to date. Our study and some previous reports suggest an association of a chromosomal aberration in gallbladder cancer [13].

The isoforms of LDH activity also increased in cases of hepatobiliary malignancy [7]. Earlier studies also suggest higher production of total LDH by tumor cells [14]. The serum level of catalase was elevated in the patients with gallbladder cancer in our study. The isoforms of SOD, such as Mn-SOD and Cu-Zn-SOD in gallbladder cancer, show differential modulation.

The present study supports the hypothesis that genetic mutation may be studied at a chromosomal level in gallbladder carcinoma. Conversely, specific chromosomal aberrations may act as markers of gallbladder cancer. Their manifestation in peripheral blood may be of diagnostic value in this disease with its poor prognosis.

## Conclusion

This study was an attempt to look into the chromosomal changes of gallbladder cancer and to identify those changes by the use of cytogenetics. Chromosomal changes in CaGB have not been done before by G- banding or using the karyotype of the chromosome of peripheral blood lymphocyte culture. This allows a look inside the complex world of genetic changes in gallbladder cancer. The simple aberrations in the form of breakage and translocation may suggest underlying genetic predisposition for development of carcinoma of the gallbladder with proper correlation. These aberrations may also act as a marker for gallbladder cancer and need further study.

## Abbreviations

CaGB: Carcinoma of the gallbladder; FBS: Fetal bovine serum; LDH: Lactate dehydrogenase; LOH: Loss of heterozygosity; NAD: Nicotinamide adenine dinucleotide; NBT: Nitroblue tetrazolium; PHA-M: Phytohemagglutinin-M; PMS: Phenozine methosulphate; RPMI: Roswell Park Memorial Institute; SOD: Superoxide dismutase; TEMED: N N N N’ tetramethyelenediamine.

## Competing interest

The authors declare that they have no competing interests.

## Authors' contributions

RD and PK wrote the article. RT, SB, RM and VKS contributed to the concept, study design, data analysis, interpretation of results and approved the final manuscript.
